# Long-Term Low Carbohydrate Diet Leads to Deleterious Metabolic Manifestations in Diabetic Mice

**DOI:** 10.1371/journal.pone.0104948

**Published:** 2014-08-29

**Authors:** Keiko Handa, Kouichi Inukai, Hirohisa Onuma, Akihiko Kudo, Fumiyuki Nakagawa, Kazue Tsugawa, Atsuko Kitahara, Rie Moriya, Kazuto Takahashi, Yoshikazu Sumitani, Toshio Hosaka, Hayato Kawakami, Seiichi Oyadomari, Hitoshi Ishida

**Affiliations:** 1 Third Department of Internal Medicine, Division of Diabetes, Endocrinology and Metabolism, Kyorin University School of Medicine, Mitaka, Tokyo, Japan; 2 Department of Anatomy, Kyorin University School of Medicine, Mitaka, Tokyo, Japan; 3 Department of Medicine, Shiga University of Medical Science, Otsu, Shiga Japan; 4 Division of Molecular Biology, Institute for Genome Research, The University of Tokushima, Kuramoto, Tokushima, Japan; Hosptial Infantil Universitario Niño Jesús, CIBEROBN, Spain

## Abstract

We investigated long-term effects of low carbohydrate diets on wild type mice, streptozotocin-injected and KKAy obese diabetic mice. These mice were pair-fed three different types of diets, standard chow (SC, C∶P∶F = 63∶15∶22), a low carbohydrate (LC, C∶P∶F = 38∶25∶37) diet and a severely carbohydrate restricted (SR, C∶P∶F = 18∶45∶37) diet for 16 weeks. Despite comparable body weights and serum lipid profiles, wild type and diabetic mice fed the low carbohydrate diets exhibited lower insulin sensitivity and this reduction was dependent on the amount of carbohydrate in the diet. When serum fatty acid compositions were investigated, monounsaturation capacity, i.e. C16:1/C16:0 and C18:1/C18:0, was impaired in all murine models fed the low carbohydrate diets, consistent with the decreased expression of hepatic stearoyl-CoA desaturase-1 (SCD1). Interestingly, both the hepatic expressions and serum levels of fibroblast growth factor 21 (FGF21), which might be related to longevity, were markedly decreased in both wild type and KKAy mice fed the SR diet. Taking into consideration that fat compositions did not differ between the LC and SR diets, we conclude that low carbohydrate diets have deleterious metabolic effects in both wild type and diabetic mice, which may explain the association between diets relatively low in carbohydrate and the elevated risk of cardiovascular events observed in clinical studies.

## Introduction

For the past decade, the identification of appropriate dietary interventions has been a source of controversy. Low carbohydrate diets have been the focus of considerable interest. Therapeutic effects of low carbohydrate diets have been extensively investigated in several clinical states, including obesity, metabolic disorders, cardiovascular events and mortality [1], [2], [3], [4], [5], [6], [7]. In short-term trials of up to one year [1], [2], [3], obese patients consuming a Mediterranean or other low carbohydrate diet exhibited more favorable conditions in terms of obesity, dyslipidemia and glycemic control. However, the conclusions drawn were limited by study design, i.e. small numbers, short periods and poor adherence to special diets. The long term safety and efficacy of a low carbohydrate diet for managing cardiovascular disease risk has yet to be determined.

Recently, in a prospective cohort study [4], low carbohydrate-high protein diets were demonstrated to be associated with increased risk of cardiovascular disease. Moreover, a carbohydrate restricted diet was reported to increase mortality [5]. Thus, clinically, detrimental effects of low carbohydrate diets have been established in recent years. However, the scientific mechanism by which a low carbohydrate diet exerts a negative effect on vascular health remains unaddressed.

To date, most rodent studies have focused on an extremely low carbohydrate, high fat diet (HFD), the so-called ketogenic diet (KD) [8], [9], [10], [11]. These mice exhibited weight loss, probably due to decreased food intake [8],[9]. In fact, a reduction in dietary carbohydrate is accompanied by an increase in dietary fat and protein. Nevertheless, it would be impossible for patients to continue consuming a KD for a prolonged period. From a practical viewpoint, a low carbohydrate diet with a moderately high fat composition should be investigated in rodent models.

In the present study, we investigated the effects of a long-term relatively low carbohydrate diet on wild type mice and two diabetic murine models, i.e. streptozotocin-induced diabetic and KKAy obese diabetic mice. It has been hypothesized that the hyperphagia-induced obesity in KKAy mice results from reductions in hypothalamic norepinephrine and dopamine [12]. As we expected to examine the effects of diets with variable carbohydrate contents on metabolic states, these mice were fed diets with three different carbohydrate compositions (63%, 38% and 18%). Unlike a KD, the fat composition was at most 37%. As giving mice low carbohydrate diets resulted in excessive caloric intake as compared to mice given standard chow, we performed a calorie-matched pair-feeding study to assure that there would be no differences in caloric intake among the three types of diets employed in this study. After 16 weeks, despite comparable body weights, mice on both LC and SR diets exhibited less insulin sensitivity, and this reduction was dependent on the amount of carbohydrate in the diet. When searching for the cause of glucose intolerance in mice given low carbohydrate diets, we discovered several deleterious metabolic manifestations, which might be due not to a moderate increase in fat composition but rather to a low carbohydrate composition. Our results support scientific evidence pertaining to the central question of why low carbohydrate diets increase the risk for cardiovascular events and/or mortality in obese or diabetic patients.

## Materials and Methods

### Ethics Statement

All animal protocol was performed according to the Guide for the Care and Use of Laboratory Animals in Kyorin University. The protocol was approved by the Committee on the Ethics of Animal Experiments of Kyorin University (Approved Number: 2014-152).

### Animals

Six-week-old male mice (C57black/6J and genetically obese KKAy) were purchased from Clea Japan, Inc. (Osaka, Japan). After a week of acclimatization, all mice were maintained individually under conditions of controlled temperature (23°C) on a 12∶12 h light-dark cycle, fed a standard rodent chow ad libitum, and had unlimited access to water. Streptozotocin (STZ) induced diabetic mice, which exhibit impairment of insulin secretion due to β-cell destruction, were prepared by two intraperitoneal injections of 0.2 ml of 50 mM sodium citrate solution (pH 4.5) containing 150 mg/kg freshly prepared STZ (Sigma-Aldrich, Co., St. Louis, MO, USA). Five days after STZ treatment, plasma glucose levels of all mice were measured and diabetes was confirmed (glucose level >300 mg/dl). Then, wild type, STZ and KKAy mice were divided into three groups (each n = 6) and calorie-matched pair-fed three different types of diets, standard chow (SC, Carbohydrate∶Protein∶Fat = 63∶15∶22), low carbohydrate diet (LC, C∶P∶F = 38∶25∶37) or a severely carbohydrate restricted diet (SR, C∶P∶F = 18∶45∶37) for 16 weeks. The precise nutrient compositions of these three diets, which were purchased from Oriental Yeast Co. (Tokyo, Japan), are presented in Table 1. We named the wild type groups WSC, WLC, and WSR, respectively, and the corresponding SC, LC and SR group were SSC, SLC, and SSR for STZ and KSC, KLC, and KSR for KKAy mice according to the type of diet.

**Table 1:** Composition of each diet.

component (g/100 g)	standard chow (SC)	lower carbo (LC)	severely carbo restriction (SR)
casein	20	30	52
cysteine	0.3	0.45	0.63
β corn starch	39.8	24.6	0
α corn starch	13.2	8.2	10.6
sucrose	10	10	10
soy oil	7	7	7
cellulose	5	5	5
AIN-93G mineral mixture	3.5	3.5	3.5
AIN-93 vitamin mixture	1	1	1
bitartrate cholin	0.25	0.25	0.25
3-butyl-hydroquinone	0.0014	0.003	0.005
lard oil	0	10	10
total	100	100	100
C∶P∶F composition (%)	63∶15∶22	38∶25∶37	18∶45∶37
total calories (kcal)	388	421	421
total intake (kcal) of wild-type mice	1061.1±3.4	1064.3±4.4	1065±5.5
total intake (kcal) of STZ mice	893.8±3.0	892±3.9	896±4.3
total intake (kcal) of KKAy mice	2151.9±9.3	2155.4±6.7	2154±8.6

### Intraperitoneal glucose tolerance test and insulin tolerance tests

For glucose tolerance tests, mice were fasted for 8 hr and 10% glucose solution was administered intraperitoneally (2 mg/g body for wild type mice, 1.5 mg/g·body for diabetic mice) as previously described [13]. Glucose measurements were conducted before injection, and at 30, 60, and 120 min after injection. For insulin tolerance tests, mice in postprandial states were intraperitoneally injected with 1.0 U/kg·body human insulin (Eli Lilly, Indianapolis, IN, USA). Glucose measurements were conducted before injection, and at 30, 60, and 90 min after injection. Plasma glucose levels were measured using a glucose analyzer (Sanwa Kagaku Kenkyusho, Co., Nagoya, Japan).

### Measurements of biomedical markers

Before sacrifice, the animals were fasted for 8 hr. Blood samples were collected by cardiac puncture using heparinized syringes and centrifuged at 12,000 rpm for 5 min. Serum lipid analyses were performed at the Skylight-Biotech Analysis Center (Akita, Japan). Hormone concentrations were measured using commercially available methods, i.e., immunoreactive insulin (IRI) was measured by radioimmunoassay (Morinaga Institute of Biological Science, Inc., Yokohama, Japan), and serum fibroblast growth factor 21 (FGF21) by Quantikine ELISA Mouse/Rat FGF-21 (R&D Systems, Minneapolis, MN, USA). Hepatic triglycerides (TG) were extracted by the methods of Bligh and Dyer [14] and analyzed using a commercially available reagent for TG (Wako Pure Chemical Industries, Ltd., Osaka, Japan). Urine was analyzed for ketones, creatinine and albumin employing a standard laboratory technique.

### Fatty acid analysis using HPLC

Fatty acids in biological samples were quantitatively measured using a modified liquid chromatography–tandem mass spectrometry (LC/MS/MS) procedure [15]. Standard solution (Myristic acid, Palmitic acid, Palmitoleic acid, Stearic acid, Oleic acid, Linoleic acid, α-Linolenic acid, γ-Linolenic acid, Dihomo-γ-Linolenic acid, Arachidonic acid, Eicosapentaenoic acid (EPA), Docosapentaenoic acid and Docosahexaenoic acid (DHA)) (Cayman Chemical Company, Ann Arbor, MI, USA) were used to obtain calibration curves. Five microliters of a serum sample or phosphate buffered saline (PBS) for the calibration curves, were transferred into glass tubes, each containing an internal solution (10 µg/mL; [^2^H_5_]-EPA and [^2^H_5_]-DHA (Cayman Chemical). Acetonitrile/6 N HCl (90/10, v/v) was added, and the tube was then capped and incubated at 100°C for 45 min. Once the tubes had reached room temperature, 200 µl of methanol/10 N NaOH (90/10, v/v) were added, followed by capping and incubation at 100°C for 45 min. After the tubes had reached room temperature, liquid/liquid extraction was performed using ethyl acetate. This upper layer was reconstituted and injected into an optimized LC/MS/MS system. LC was performed using an ACQUITY UPLC (Waters, Milford, MA), and an API4000 triple quadrupole tandem mass spectrometer (AB Sciex, Foster City, CA, USA) was used as a detector. An analytical column, YMC-Triart C18 (2.0 mm×100 mm, particle size 1.9 µm) (YMC Co.,Ltd., Kyoto, Japan), was used for separating the fatty acids from each other. For operation of the API4000, atmospheric pressure chemical ionization in negative ionization and selected reaction monitoring mode was applied.

### Quantative analysis of hepatic mRNA and proteins

Mice were fasted for 8 hr before tissue harvesting. The mice were killed by cervical dislocation, and liver and epididymal fat tissues were rapidly removed and weighed. Hepatic total RNA was isolated with Isogen (Nippon Gene, Tokyo, Japan). To quantitatively analyze mRNA for the indicated gene, we conducted real-time PCR using an ABI PRISM Model 7000 (Applied Biosystems, Foster City, CA, USA) according to the manufacturer's instructions. The primer sets and probes for mouse insulin substrate 1 (IRS1), IRS2, Forkhead box protein O1 (FoxO1), phosphoenolpyruvate carboxylase (PEPCK), glucose 6 phosphatase (G6Pase), fatty acid synthase (FAS), peroxisome proliferator activator receptor γ (PPARγ), PPARα, signal transducer and activator of transcription 3 (STAT3), interleukin 6 receptor (IL6R), leptin receptor, uncoupling protein 2, adiponectin receptor 1(adipoR1), sirtuin 1, FGF21, peroxisome proliferator activator recepto γ coactivator α (PGC1α), stearoyl-CoA desaturase-1 (SCD1) and elongation of long chain fatty acid member 6 (Elovl6) were purchased from Applied Biosystems. Western blot analysis was performed as previously described [16]. Briefly, liver samples from mice were homogenized in lysis-buffer (1% Triton/PBS) and centrifuged at 14,000× g for 10 min at 4°C. Supernatants including tissue protein extracts were resolved on 10% SDS-PAGE gel, followed by electrophoretic transfer to a nitrocellulose membrane. After blotting with a polyclonal antibody against mouse SCD-1 (Cell Signaling Technology, Inc., Danvers, MA, USA) or mouse PGC1α (Abcam Inc., Cambridge, MA), detection was performed using an ECL chemiluminescent kit (GE Healthcare Life Sciences, Buckinghamshire, UK) according to the manufacturer's instructions. Quantitations were performed using a Molecular Imager (Bio-Rad Laboratories, Hercules, CA, USA). As an internal control, we performed western blot using anti β-actin antibody (Sigma-Aldrich).

### Immunohistochemistry for hepatic oxidative stress markers (4-HNE and 8-OHG)

The slices of liver tissue were immersed in 4% paraformaldehyde-PBS at 4°C overnight, and then embedded in paraffin employing routine procedures. Deparaffinized sections (4 µm thick) were rehydrated and used for immunohistochemical staining as previously described [17]. Briefly, the sections were first immersed in Antigen Unmasking Solution pH. 6.0 (Vector Laboratories Inc., Burlingame, CA, USA) and the temperature was kept above 95°C for 30 min by wet-autoclaving. The antigen-retrieved sections were treated with 5% donkey serum-PBS, then incubated with the primary antibodies for 1 hr at room temperature (RT): rabbit anti-4-hydroxynonenal (4-HNE) polyclonal antiserum (HNE11-S; 1∶5,000 dilution; Alpha Diagnostic International, Inc., San Antonio, TX, USA), goat anti-8-hydroxyguanosine (8-OHG) antiserum (8OHG12-S; 1∶2,000 dilution; Alpha Diagnostic International, Inc.). We included a negative control (lacking primary antibody) for each immunohistochemical analysis. The sections were successively treated with ImmPRESS Reagent (Vector Laboratories) as a secondary antibody (anti-rabbit IgG and anti-goat IgG, respectively) for 30 min. Then, the sections were incubated with diaminobenzidine (DAB) solution (Wako Pure Chemical Industries, Ltd.) for 10 min at RT to detect the peroxidase enzyme activity. Finally, sections were counterstained with hematoxylin. Three optical fields of sections from each animal (n = 3) were randomly chosen and photomicrographed. The staining intensity was quantitated using Image J software (National Institute of Health, Bethesda, MD, USA). Briefly, color images were first subjected to color deconvolution [18] using G. Landini's plugin to obtain separate images for DAB and hematoxylin in gray scale. We measured the mean optical density of DAB staining in the cytoplasm for 4-HNE and that in the area of nuclei for 8-OHG, applying binary mask images of nuclei created from hematoxylin images, and evaluated the staining intensity.

### Statistical analysis

Data are presented as means ± SEs. Log transformation of continuous variables was used when needed to satisfy distributional requirements for parametric tests. Comparisons between two groups were made with Student's t-test. For comparisons among three groups, one-way ANOVA was used with the Tukey test. A *P* value<0.05 was considered statistically significant. Statistical analyses were performed using Stat View software (Version 5.01; SAS Institute, Cary, NC, USA).

## Results

### Glucose tolerance and insulin sensitivity depend on the dietary carbohydrate component

**Figure 1:**
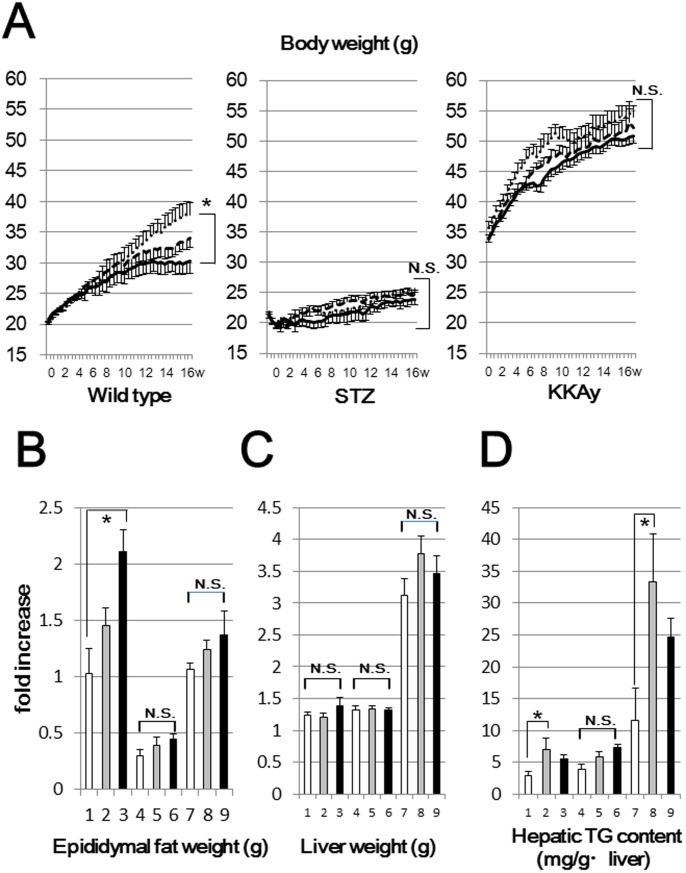
Body weight changes, liver weight, epididymal fat weight and hepatic TG contents in each group (n = 6). (A) Wild type (left panel), STZ (middle panel) and KKAy (right panel) mice were pair-fed a standard chow (SC diet, solid lines), a low carbohydrate (LC diet, dashed lines) or a severely carbohydrate restricted diet (SR diet, dotted lines) for 16 weeks. Liver weight (B), epididymal fat weight (C) and hepatic TG contents (D) were analyzed in each group at the end of the study (line 1:WSC, line 2:WLC, line 3:WSR, line 4:SSC, line 5:SLC, line 6:SSR, line 7:KSC, line 8:KLC, line 9:KSR). *p<0.05 (vs corresponding mice fed the SC diet).

In a preliminary experiment, ad libitum consumption was monitored for 1 week and no significant differences in food intakes were observed among mice fed the 3 diets. As our goal was to investigate the effects of these 3 diets with similar caloric intakes, we restricted the food intakes of mice fed the LC and SR diets, which have higher caloric densities than the SC diet (Table 1). The diets were provided daily at 6 p.m. and the amounts of the LC and SR diets were determined to be the same calorically as the mean of the calories consumed by the mice fed the SC diet on the previous day. Under these conditions, mice were pair-fed the 3 different diets for 16 weeks. The mean of total calories during the 16 weeks of this study, i.e. that consumed by each mouse, is shown at the bottom of Table 1. Fig. 1A shows the body weight changes in all groups throughout the study. Despite the food intake restrictions in WSR, these mice exhibited significantly increased weights as compared with WSC. On the other hand, no significant differences in body weights were seen in the STZ and KKAy diabetic mice. KKAy mice have such abundant subcutaneous and mesenteric adipose tissues that we could not weigh them accurately. Thus, we weighed epididymal fat, which likely represents the fat accumulation in WAT (Fig. 1B). In WSR, epididymal fat accumulation was significantly increased, suggesting weight gain to be attributable to overall fat accumulation. In STZ and KKAy mice, there were no significant differences in fat weights among the three diets. In these mice, the downregulation of insulin signaling due to insulin deficiency or insulin resistance may blunt fatty acid synthesis, which may explain why the body or fat weight changes in these diabetic mice did not reach statistical significance. As shown in Fig. 1C and 1D, the analysis of hepatic TG contents revealed increased ectopic fat accumulations in mice fed the LC diet, but these increases did not result in significant hepatic weight gains.

As shown in Table 2, basic metabolic biomarkers were analyzed. The fasting plasma glucose and fasting IRI levels were significantly higher in WSR and KSR than in WSC and KSC, respectively, indicating deterioration of insulin sensitivity in these mice. In contrast, no significant differences were observed in postprandial plasma glucose levels, though these levels tended to be higher in mice receiving the SC diet than in those on the SR diet. Total cholesterol (T-chol), low density lipoprotein-chol, high density lipoprotein-chol and TG levels differed minimally among the three diets. Urinary ketone/creatinine concentrations (mmol/gCre) were also analyzed. While only diabetic mice given the SR diet showed a significantly higher ketone level, the ketogenic effects of the SR diet, i.e., moderately increased fat and severely restricted carbohydrate, were very small as compared with those seen in STZ mice, i.e., in a state of insulin deficiency. Urinary albumin/creatinine ratios were significantly elevated in diabetic mice fed the LC diet. When dietary carbohydrate is reduced, the protein composition inevitably increases, resulting in elevated urinary albumin.

**Table 2:** Biochemical parameters of each group.

	FPG(mg/dl)	PPG(mg/dl)	FIRI(ng/ml)	Tcho(mg/dl)	LDLc(mg/dl)	HDLc(mg/dl)	TG(mg/dl)	keton(mmol/gCr)	albumin(mg/gCr)
WSC	79.8±6.6	203.5±16.4	1.06±0.30	115.7±8.4	17.1±1.8	92.8±6.4	39.2±2.8	0.11±0.03	0.026±0.010
WLC	109.8±15.5	192.8±16.4	1.24±0.05	127.1±1.4	23.3±0.8	100.5±1.9	21.6±2.3	0.10±0.03	0.031±0.010
WSR	134.4±6.8*	156.8±17.9	1.77±0.13*	122.0±3.6	17.4±1.4	100.1±1.0	39.9±4.1	0.15±0.03	0.033±0.020
SSC	135.8±8.0	305.3±28.5	0.58±0.17	99.3±15.3	11.8±1.7	78.5±11.3	61.5±19.3	40.5±29.8	0.151±0.013
SLC	145.4±8.9	240.5±10.3	1.40±0.21	118.9±9.5	16.5±2.6	88.4±4.1	87.7±26.9	50.5±37.3	0.618±0.261*
SSR	110.8±6.4	235.0±21.5	1.07±0.17	103.6±3.1	9.3±0.7	82.7±3.0	130.9±20.0	168.7+88.0*	0.337±0.023
KSC	85.5±8.5	522.0±51.9	23.5±11.6	129.3±9.2	14.4±0.8	116.8±16.2	179.3±4.2	0.17±0.01	3.526±1.330
KLC	80.2±8.0	579.3±37.0	55.5±9.5	158.5±9.6	11.8±1.0	131.6±8.4	229.3±11.4	0.25±0.04	9.859±0.927*
KSR	140.7±25.2*	462.8±56.9	141.0±29.1*	139.0±14.5	16.8±5.2	111.8±8.2	137.6±3.8	0.51±0.11*	5.772±2.852

*p<0.05 (vs corresponding mice fed the SC diet).

**Figure 2:**
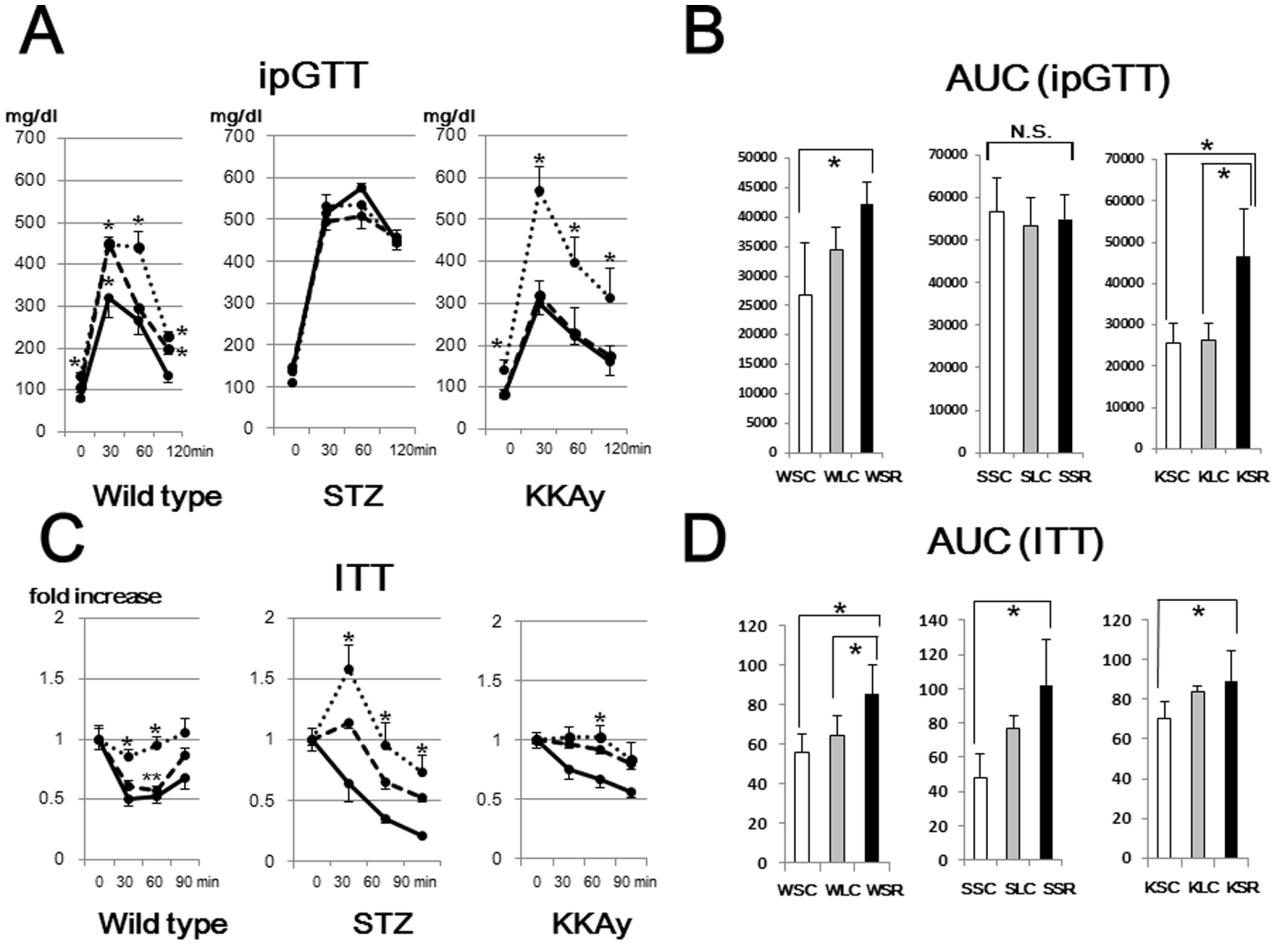
Intraperitoneal glucose tolerance test (ipGTT) and insulin tolerance test (ITT). (A) Mice fed the SC diet (solid lines), LC diet (dashed lines) and SR diet (dotted lines) were fasted for 8 hr and glucose solution was administered intraperitoneally (2 mg/g body for wild type mice, 1.5 mg/g·body for diabetic mice). (B) Area under the curve (AUC) was calculated to measure the degree of the glucose tolerance impairment. (C) Mice in postprandial states were intraperitoneally injected with 1.0 U/kg·body human insulin. (D) AUC was calculated to measure the degree of the insulin tolerance impairment. *p<0.05 (vs corresponding mice fed the SC diet). **p<0.05 (vs corresponding mice fed the SR diet).

In an intraperitoneal glucose tolerance test, WSR and KSR exhibited marked glucose intolerance as compared with WSC and KSC, respectively, mice (Fig. 2A, 2B). In STZ mice, there were no significant differences among the three diets, suggesting the deterioration of glucose tolerance due to insulin deficiency to be so striking that dietary effects on glucose intolerance might be negligible. In the insulin tolerance test (Fig. 2C, 2D), insulin sensitivity was decreased in mice fed the SR diet as compared to those given the SC diet, and this reduction was dependent on the amount of carbohydrate in the diet. These results suggest that a low carbohydrate diet exerts detrimental effects on insulin sensitivity in both diabetic and nondiabetic mice.

### Screening for the cause of glucose intolerance by examining hepatic transcriptional levels of various genes in mice fed the SC and SR diets

**Figure 3:**
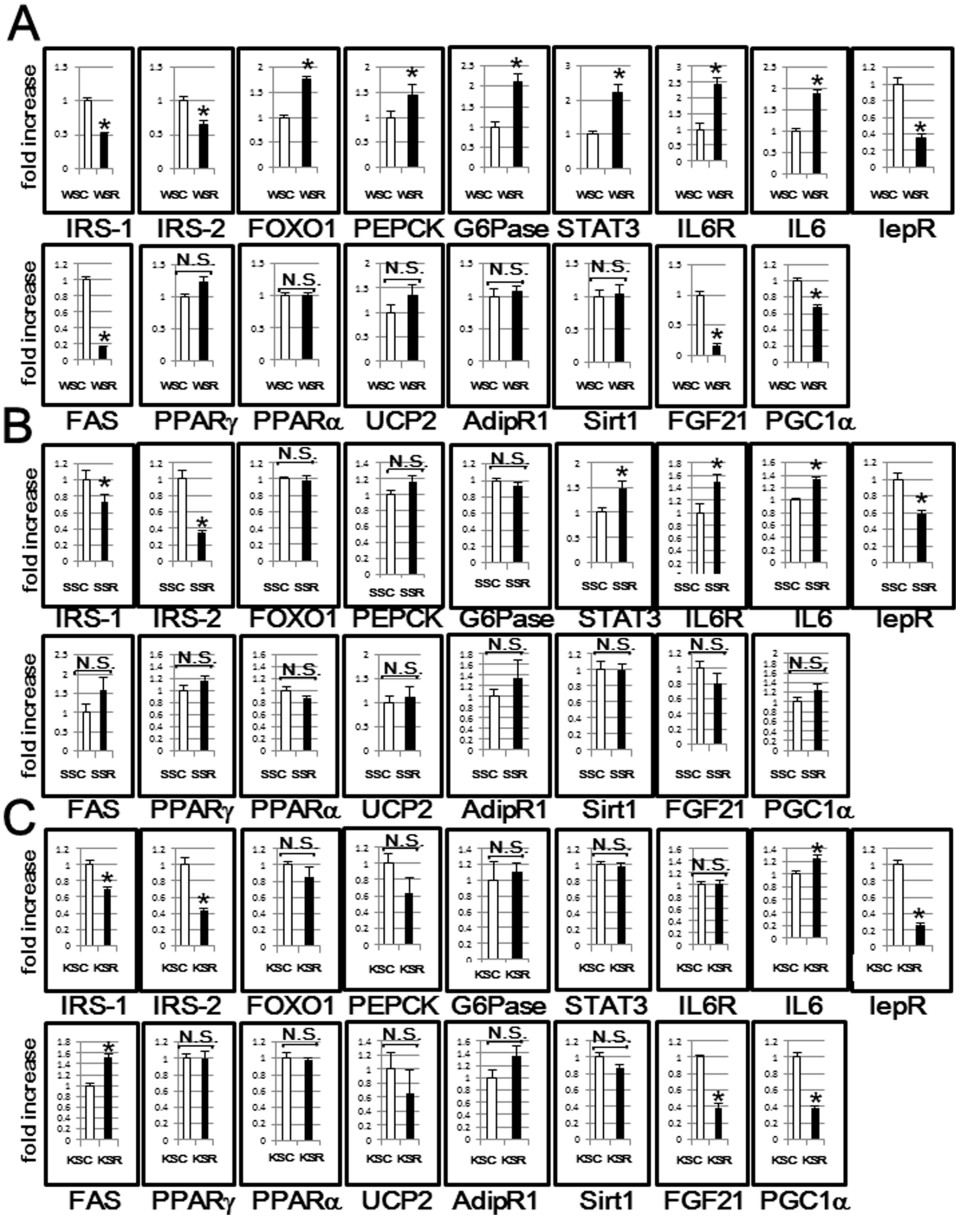
Screening for the cause of glucose intolerance by examining hepatic transcriptional levels of various genes in wild type, STZ and KKAy mice fed the SC or SR diet. Hepatic mRNA expressions of each of the genes in wild type (A), STZ (B) and KKAy (C) mice fed the SC or SR diet were evaluated by RT-PCR, and presented as the fold increases compared to mice fed the SR diet. *p<0.05 (SC fed vs. SR fed mice).

Next, in order to screen for the causes of glucose intolerance in these mice, the hepatic expressions of genes related to glucose and lipid metabolism in mice fed the SC and SR diets were analyzed using real time PCR. The results for wild type mice (WSC vs. WSR) are presented in Fig. 3A. As to insulin signaling molecules, the decreases in IRS-1 and -2 expressions and the increase in FoxO1 expression were significant. The upregulations of gluconeogenesis related enzymes, i.e., PEPCK and G6Pase, might account for the higher FPG in WSR mice. In contrast, hepatic STAT3, which plays a role in the inhibition of gluconeogenesis-related enzymes, was increased. As IL6R and IL6 expressions were both increased, the activation of STAT3 signaling might not be due to activation of the leptin signal, or rather to activation of IL6 signaling. These results suggest that the STAT3 signal compensates for reduced insulin signaling in wild type mice. As to lipid metabolism, FAS was inhibited, which might be attributable to the negative feedback system followed by TG accumulation, as previously reported [19]. Whlie results similar to those of wild type mice were obtained for some hepatic gene expressions, others in diabetic mice did not differ significantly between mice fed the SC and SR diets (Fig. 3B, 3C), in contrast to those in wild type mice. In particular, the significant elevations of gluconeogenesis-related enzymes, observed in WSR as compared with WSC, were minimal in diabetic mice. These results suggest that the up-regulation of hepatic gluconeogenesis observed in WSR, may not be due simply due to the subsequent phenomenon resulting from hepatic insulin resistance, instead being attributable to be required compensatory mechanism in response to glucose deprivation. In fact, the up-regulation of hepatic gluconeogenesis was blunted in diabetic mice, which have no need to prevent hypoglycemia. We searched for further possible explanations of reduced glucose intolerance and, interestingly, found the expression of hepatic FGF21, which has a role in ameliorating insulin resistance [20], to be markedly decreased in both wild type and KKAy mice.

### Both the hepatic expression and serum levels of FGF21 were markedly decreased in WSR and KSR mice

**Figure 4:**
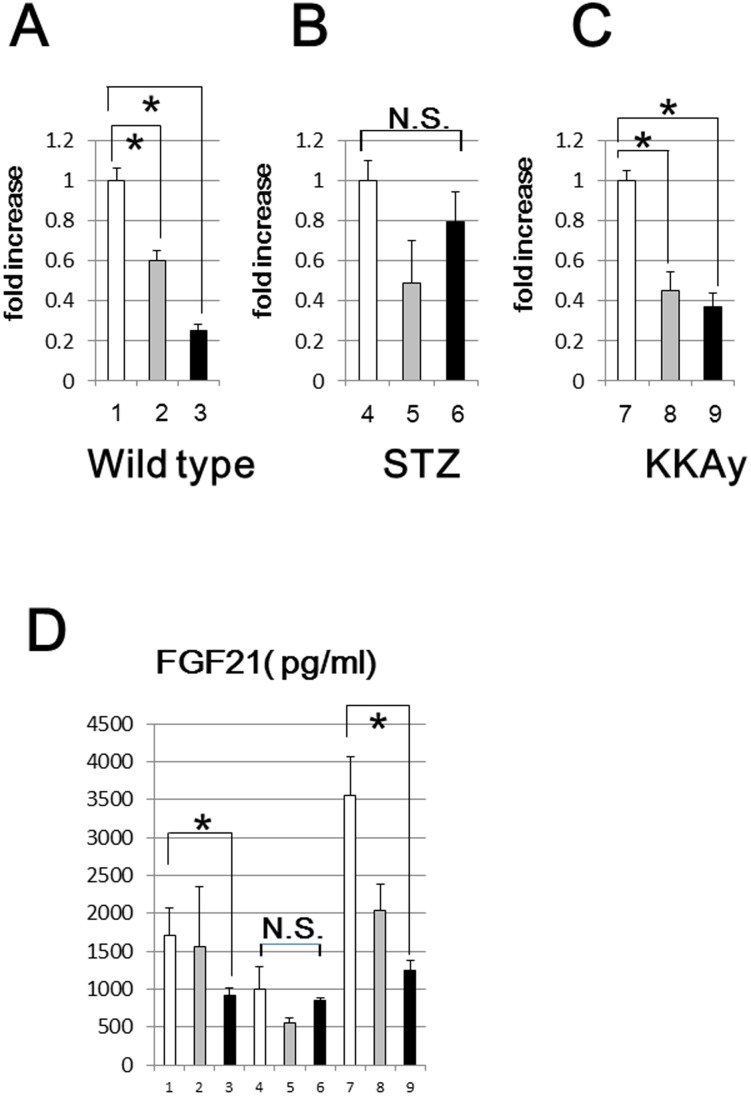
Hepatic mRNA expression and serum levels of FGF21. FGF21 mRNA levels of wild type (A), STZ (B) and KKAy (C) mice were analyzed by quantitative real time PCR. Serum FGF21 levels (D) were measured using an ELISA kit (line 1:WSC, line 2:WLC, line 3:WSR, line 4:SSC, line 5:SLC, line 6:SSR, line 7:KSC, line 8:KLC, line 9:KSR mice). *p<0.05 (vs corresponding mice fed the SC diet).

**Figure 5:**
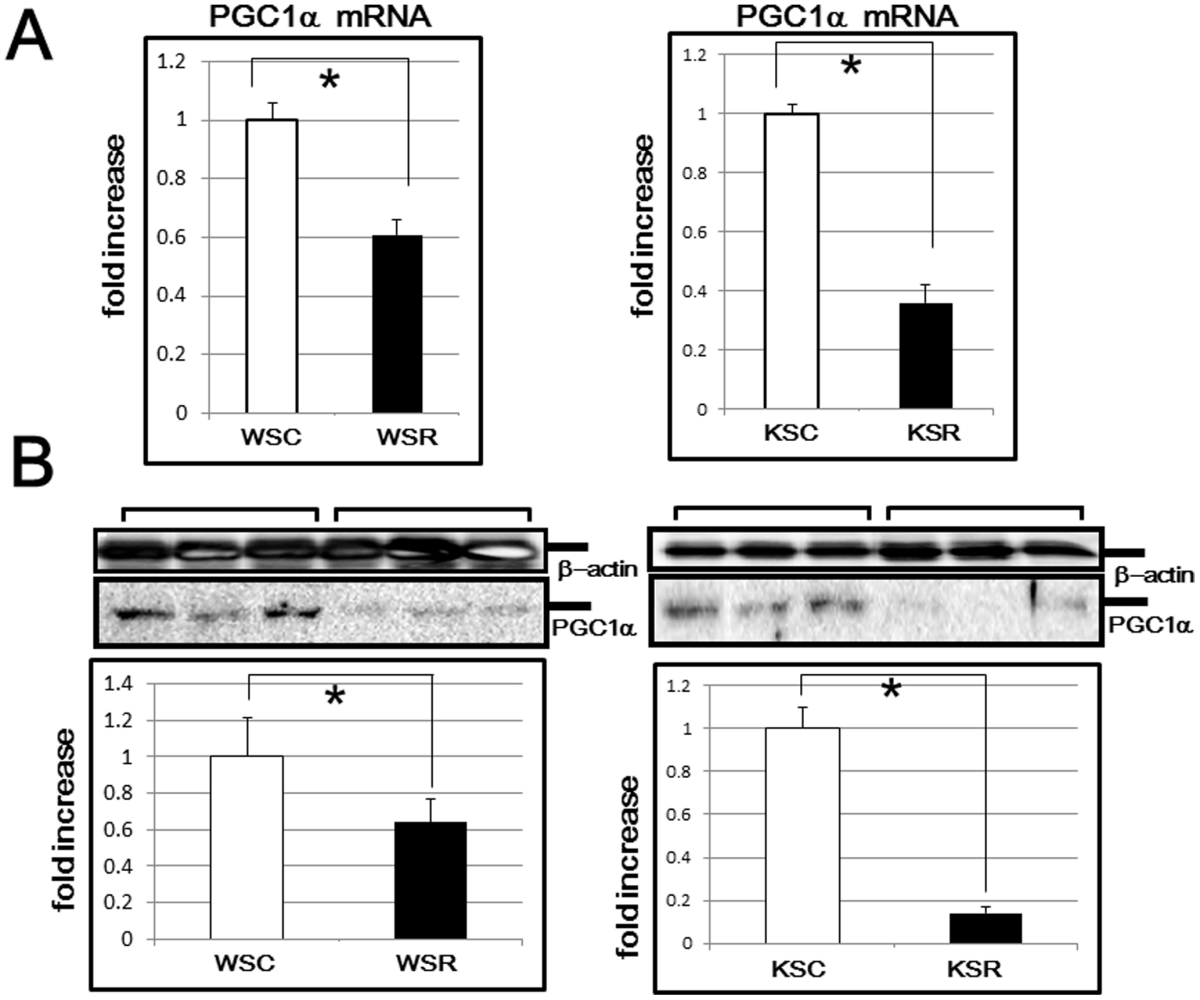
mRNA and protein levels of PGC1α in WAT. (A) PGC1α mRNA levels of wild type and KKAy mice were analyzed by RT-PCR. *p<0.05 (vs corresponding mice fed the SC diet). (B) PGC1α protein levels of wild type and KKAy mice were analyzed by western blotting. In the middle panels, representative data (three samples for each group) are presented. In the lower panels, each column shows the mean ± S.E. obtained from 6 mice (white bar: mice fed the SC diet, black bar: mice fed the SR diet). Upper panels show the internal control using anti-β actin antibody. *p<0.05 (vs corresponding mice fed the SC diet).

We investigated the hepatic mRNA levels of FGF21 in all groups of mice in detail. As shown in Fig. 4A–C, in wild type and KKAy mice, hepatic FGF21 expressions were decreased, depending on the amount of carbohydrate in the diet, while no statistically significant difference was observed in STZ mice. To confirm this result, we further examined the serum levels of FGF21 by ELISA and obtained results consistent with those of hepatic transcriptional expression (Fig. 4D). These findings might explain, at least in part, the glucose intolerance in WSR and KSR in comparison with WSC and KSC mice, respectively. Next, we investigated the downstream target of FGF21 and found that PGC1α, which has crucial roles in energy expenditure in adipose tissues, was markedly reduced in both wild type and KKAy mice fed the SR diet at both the transcriptional (Fig. 5A) and the translational level (Fig. 5B). These results might explain the greater weight gain in WSR than WSC.

### Decreased expression of stearoyl CoA desaturase 1 (SCD-1) in mice fed low carbohydrate diets

**Figure 6:**
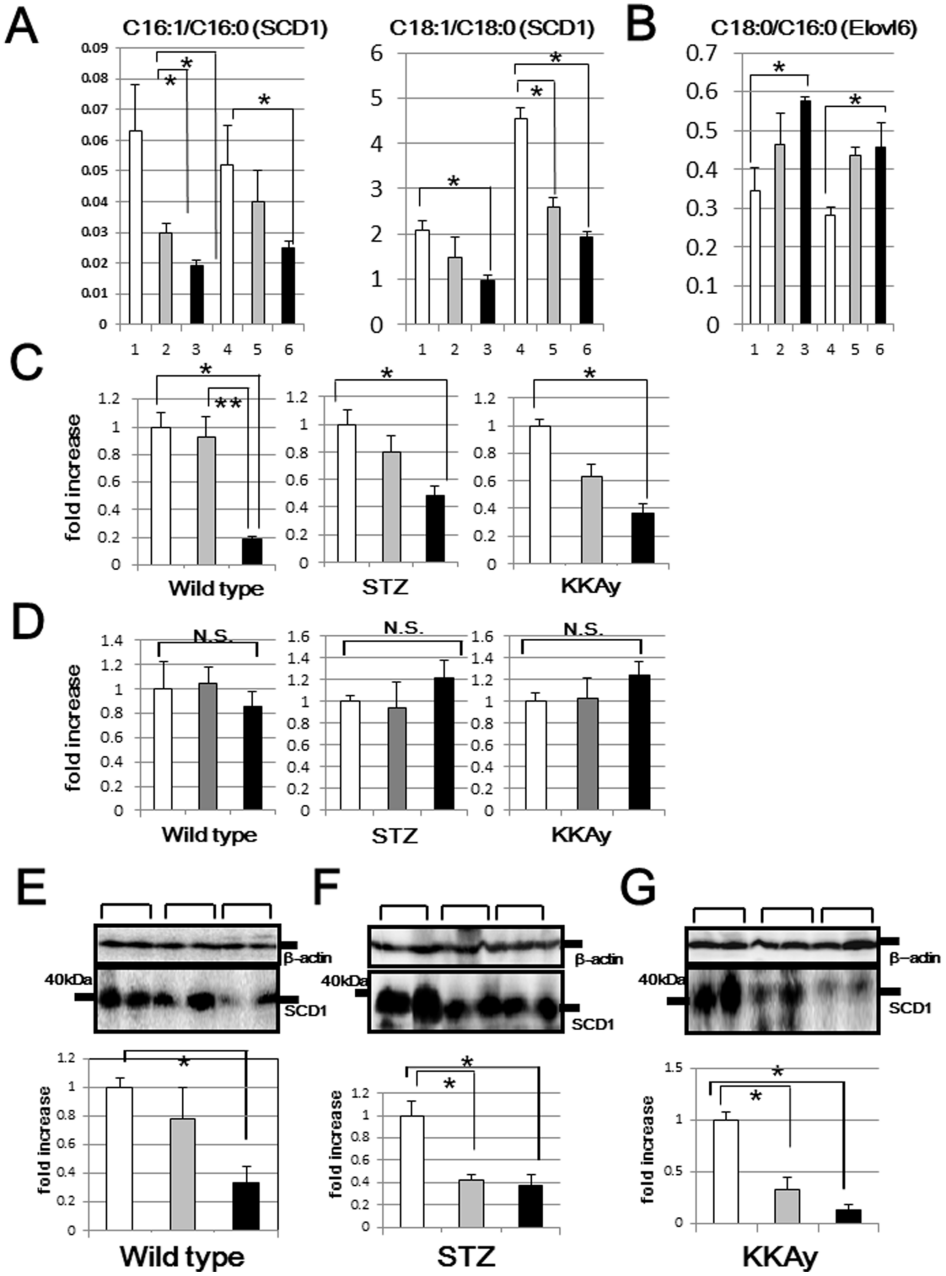
Hepatic SCD1 mRNA and protein levels of wild type, STZ and KKAy mice. The C16:1/C16:0, C18:1/C18:0 (A) and C18:0/C16:0 (B) ratios were calculated, based on data obtained from the serum fatty acid analysis (line 1:WSC, line 2:WLC, line 3:WSR, line 4:KSC, line 5:KLC, line 6:KSR). SCD1 (C) and Elovl6 (D) mRNA levels of all murine models were analyzed by quantitative real time PCR (white bar: mice fed the SC diet, gray bar: mice fed the LC diet, black bar: mice fed the SR diet). Hepatic SCD1 protein levels of wild type (E), STZ (F) and KKAy (G) mice were analyzed by western blotting. In the middle panels, representative data (two samples for each group) are presented. In the lower panels, each column shows the mean ± S.E. obtained from 6 mice (white bar: mice fed the SC diet, gray bar: mice fed the LC diet, black bar: mice fed the SR diet). Upper panels show the internal control using anti-β actin antibody. *p<0.05 (vs corresponding mice fed the SC diet). **p<0.05 (vs corresponding mice fed the SR diet).

**Figure 7:**
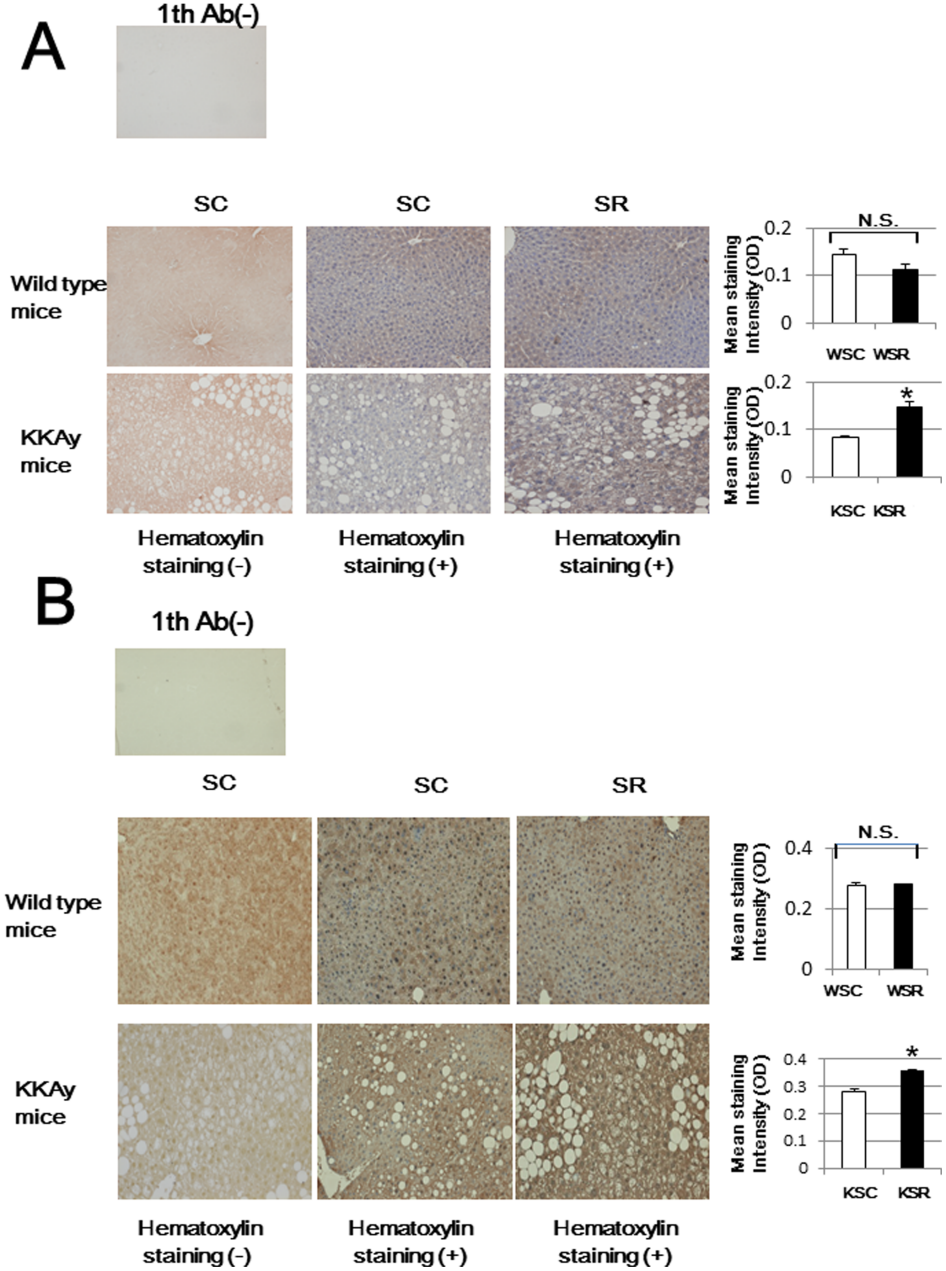
Immunohistochemistry for 4-HNE and 8-OHG. Three optical fields of sections from each animal (n = 3) were randomly chosen and photomicrographed. Original magnification is ×50. The staining intensity was quantitated using Image J. Color images were first subjected to color deconvolution using G. Landini's plugin to obtain separate images of DAB and hematoxylin in gray scale. We measured the mean optical density of DAB staining in the cytoplasm for 4-HNE (A) and that in the area of nuclei for 8-OHG (B), applying binary mask images of nuclei created from hematoxylin images, and evaluated the staining intensity. Upper panels are negative controls, lacking primary antibody. One representative image of wild type (middle panels) and KKAy (lower panels) mice, fed the SC diet (left panels: without hematoxylin staining, middle panels: with hematoxylin staining) and the SR diet (right panels: with hematoxylin staining) is presented. Right bar graphs show staining intensity averages. *p<0.05 (KSC vs. KSR mice).

We next analyzed serum fatty acid compositions by using LC/MS/MS. As shown in Table 3, monounsaturated fatty acids, i.e., palmitoleic acid (C16:1) and oleic acid (C18:1), were strikingly decreased in mice fed the SC diet as compared to those in mice receiving the SR diet. Thus, we calculated C16:1/C16:0 and C18:1/C18:0 ratios to clarify monounsaturation activity. As shown in Fig. 6A, monounsaturation reactions were impaired in mice fed low carbohydrate diets, while the elongation reaction (C18:0/C16:0) was conversely increased (Fig. 6B). Then, transcriptional and translational expressions of hepatic SCD1, an enzyme that catalyzes the synthesis of monounsaturated fatty acids, were analyzed (Fig. 6C, Fig. 6E–G). Consistent with the decreased C16:1/C16:0 and C18:1/C18:0 ratios, the expression of hepatic SCD1 in both wild type and diabetic mice also decreased, and this reduction was dependent on the amount of carbohydrate in the diet. In contrast, there were no differences in transcriptional levels of hepatic Elovl6 (Fig. 6D), a rate-limiting enzyme catalyzing the elongation of saturated and monounsaturated fatty acids, among mice fed the 3 diets. Considering that oleic acid has a crucial role in reducing oxidative stress [21], we confirmed the hepatic accumulation of oxidative stress in a histochemical study. We quantitatively evaluated immunostaining for 4-HNE in the cytoplasm and 8-OHG in nuclei as oxidative stress markers. As shown in Fig. 7, while neither 4-HNE nor 8-OHG accumulations were found to differ between WSC and WSR, there were significant differences between KSC and KSR, which partially explains the marked increase in insulin resistance in KSR mice. Histologically, KKAy mice had fatty livers without inflammatory cellular infiltrations. Though the reason for KKAy mice showing these differences in reactive oxygen species (ROS) accumulation remains unknown, fatty liver observed in KKAy mice, which are vulnerable to oxidative stress, may have accelerated the hepatic ROS accumulation only in KSR mice.

**Table 3:** Fatty acid composition of each group (mg/mL).

Fatty acid composition		Wild type (SC)	Wild type (LC)	Wild type (SR)	KKAy (SC)	KKAy (LC)	KKAy (SR)
Palmitic acid	C16:0	777.1±103.6	692.1±48.4	444.6±82.3	1122.8±148.0	1226.9±188.4	1055.8±128.9
Palmitoleic acid	C16:1	51.3±18.8	20.7±2.8	8.3±0.9	61.6±29.8	45.8±7.1	26.0±2.3
Stearic acid	C18:0	275.6±82.3	315.4±52.2	254.4±44.0	311.8±22.7	524.5±52.5	486.2±105.2
Oleic acid	C18:1 n = 9	544.2±109.6	434.6±68.0	233.41±15.0	1424.1±179.8	1336.5±65.1	930.5±161.3
Linoleic acid	C18:2 n = 6	863.2±121.0	826.1±57.9	597.6±90.8	1091.2±63.0	1326.4±103.5	1131.6±186.7
α-Linoleic acid	C18:3 n = 3	16.6±3.9	10.8±1.4	3.8±0.6	34.0±7.5	38.7±6.4	19.0±1.9
γ-Linoleic acid	C18:3 n = 6	8.9±2.8	10.0±1.2	7.2±3.9	10.4±0.4	13.6±2.0	11.1±1.4
Arachidonic acid	C20:4 n = 6	619.6±98.8	732.5±2.8	550.4±68.8	651.4±27.7	815.6±179.8	754.2±73.1
Eicosapentaenoic acid	C20:5 n = 3	23.6±60.1	18.7±2.7	15.1±2.1	40.1±4.1	31.4±3.0	29.1±4.5
Docosahexaenoic acid	C22:6 n = 3	252.1±39.3	255.6±10.5	214.0±21.2	214.5±10.3	268.9±52.3	286.2±53.1
Total fatty acid		3556.8±614.3	3404.7±109.4	2400.9±309.2	5098.2±454.0	5769.9±558.1	4865.7±687.6

## Discussion

In this study, we prepared 3 types of diets, i.e. a standard chow, a relatively low carbohydrate and a severely carbohydrate restricted diet. The SC diet, containing 63% carbohydrate, corresponds to a high carbohydrate/low fat diet, which has an established record of safety and efficacy. Thus, an evidence-based recommendation for high carbohydrate (≧55%) nutrition therapy for individuals with diabetes exists in most countries, including Japan [22]. The C∶P∶F composition of the LC diet, i.e. moderately reduced carbohydrate content (35∼40%)/moderately high fat diet (35∼40%), is a Mediterranean-like diet, which is among the most popular low carbohydrate diets in western societies [23]. In addition, we prepared a SR diet to investigate how such a diet influences human health. In previous studies, mice fed a KD or a HFD exhibited marked reductions in body weight [8], [9]. In a human study as well, low carbohydrate diets were effective for weight loss [1], which might be attributable to the decreased intake and appetite loss associated with low carbohydrate diets [6]. However, these effects lasted only for 6 months [1], probably because patients adapted to the low carbohydrate diets and their appetites were restored. Therefore, in such rodent experiments and human intervention studies, it is not possible to distinguish various effects of the low carbohydrate diet from those of the associated decreased calorie intakes. In the present study, we devised the SR diet to have a high protein instead of a high fat content in order to avoid appetite reduction and weight loss. In addition, we employed pair-feeding, in which each mouse had the same caloric intake as its counterpart. Thus, our experiments precisely reflect the actual effects of a low carbohydrate diet.

As the aim of this study was simply to investigate the effects of different macronutrients on biological phenotypes in wild type and diabetic mice, we could not prepare corresponding control mice, i.e. STZ mice versus PBS injected mice, or KKAy versus KK mice. Thus, our observations do not allow us to draw conclusions about the differences between animal models. In a preliminary experiment, we prepared STZ mice by a conventional method [24]. However, STZ mice fed the SR diet (SSR mice) did not survive for more than a week after injection, which might be attributable to severe ketoacidosis. Thus, we changed the protocol and injected a smaller amount of STZ as mentioned in the Methods. With this modification, the STZ mice used in our study exhibited less plasma glucose elevation and higher fasting insulin levels than the STZ mice previously described [24], indicating that these mice are not like type 1 diabetes models, instead resembling type 2 diabetes models with β cell exhaustion.

We unexpectedly discovered that both hepatic expressions and serum levels of FGF21 were suppressed in wild type and KKAy mice fed the low carbohydrate diets, and that these reductions were dependent on the amount of carbohydrate in the diet. As FGF21 positively regulates the expressions of thermogenesis-related genes, i.e., UCP-1 and PGC1α, in white adipose tissues [25], these decreases in FGF21 levels are likely to be the main cause of weight gain in mice fed low carbohydrate diets. In very recent studies [11], [20], [26], FGF21 was demonstrated to have a crucial role in enhancing insulin sensitivity. FGF21 administration to obese mice resulted in improvements in hepatic insulin sensitivity, which may be attributable, at least in part, to increased energy expenditure in the liver and white adipose tissue. Thus, markedly decreased serum FGF21 levels likely explain their insulin intolerance. However, in these studies, HFD fed mice exhibited higher serum levels of FGF21 [27], which would appear to be incompatible with our present results. We can explain this contradiction as follows: both hepatic expression and circulating levels of FGF21 are known to be strongly up-regulated by fasting or ketogenesis induced by HFD [10]. In our study, wild type and KKAy mice fed the LC or SR diet did not manifest ketogenesis, because fat compositions (37%) in these diets are not as high as in HFD. Thus, FGF21 expression might not be stimulated under these non-ketotic conditions. In contrast, the results obtained in STZ mice were rather ambiguous, i.e. no significant difference in FGF21 expressions among mice fed the 3 diets, possibly reflecting moderate ketogenesis in STZ mice. Though hepatic FGF21 was previously reported to be induced by acute exercise [28] or endoplasmic reticulum stress [29], we could not identify the mechanism by which FGF21 was down-regulated by long-term feeding of a low carbohydrate diet. In a very recent study [30], βKlotho, known as a longevity-promoting gene, was demonstrated to be essential for the beneficial metabolic actions of FGF21. These two proteins were revealed to work cooperatively. Moreover, transgenic overexpression of FGF21 markedly extends lifespans in mice without reducing food intake [31]. These findings raise the possibility that FGF21 can be expected to extend lifespan. Therefore, the evidence obtained in this study strongly supports those of the clinical study [5], in which a low carbohydrate diet increased mortality.

We further assessed the serum compositions of fatty acids, because fatty acids were reported to be associated with insulin sensitivity [32]. In this analysis, the conversion from saturated fatty acid to monounsaturated fatty acid, corresponding to hepatic SCD1 activity was decreased in mice fed the low carbohydrate diets, and this reduction was dependent on the amount of carbohydrate in the diet. The present study is, to our knowledge, the first to directly confirm fatty acid composition changes in rodents, while the decreased SCD1 expression was previously reported in obese humans [33]. Though the precise mechanism of hepatic SCD1 suppression remains unknown, several possibilities have been raised. For example, the transcriptional downregulation of SCD1 was suggested to be associated with fatty liver [34]. A previous noteworthy study [35] showed murine hepatic SCD1 expression to be induced by consuming a fat-free, high carbohydrate diet, which is consistent with our results. Taken together, our results support the idea that dietary carbohydrates regulate the expression of hepatic SCD1. Intake of monounsaturated fatty acids has been reported to reduce oxidative stress and insulin resistance [36]. In a very recent *in vitro* study, incubation with saturated fatty acids induced marked ROS accumulation in rat hepatocytes, while incubation with oleic acids did not [37]. Thus, the decreased SCD1 expression, i.e., C16:1/C16:0 ratio, observed in mice fed the low carbohydrate diets may partially be explained by the hepatic accumulation of oxidative stress observed in our immunohistochemical analysis. Numerous clinical studies have shown that a Mediterranean diet, which involves the use of olive oil as the principle fat component, protects against cardiovascular diseases [38]. Taking into consideration that the C∶P∶F composition of the Mediterranean diet is similar to that of the LC diet, the addition of olive oil may exert a beneficial effect by reversing the major disadvantage of this popular diet, which may disturb the production of monounsaturated fatty acids in the liver.

One of the limitations of our study is that we cannot rule out the possibility that our observations may have been affected by the high-protein diets provided, since protein and carbohydrate proportions are inversely proportional. In a very recent rodent study, the carbohydrate/protein ratio, not calorie intake, was found to determine cardiometabolic health, aging and longevity in mice [39]. Though our findings do not allow us to identify which macronutrient, i.e., low carbohydrate or high protein, is detrimental to health, it is noteworthy that our findings shed light on the cardiometabolic phenotypes affected by the carbojydrate/protein ratio.

We investigated the long-term effects of a low carbohydrate diet on diabetic murine models. These mice, when fed the low carbohydrate diet, exhibited glucose intolerance, decreased serum levels of FGF21 and also decreased expression of hepatic SCD1. All of these reductions were dependent on the amount of carbohydrate in the diet. Notably, these manifestations were unrelated to weight regulation in diabetic mice. To our knowledge, this is the first report indicating that a low carbohydrate diet leads to deleterious metabolic manifestations in diabetic mice, which may explain the close link between such diets and the high risks for cardiovascular events and morbidity observed in clinical settings.
