# Correction: Long-Term Low Carbohydrate Diet Leads to Deleterious Metabolic Manifestations in Diabetic Mice

**DOI:** 10.1371/journal.pone.0155751

**Published:** 2016-05-16

**Authors:** Keiko Handa, Kouichi Inukai, Hirohisa Onuma, Akihiko Kudo, Fumiyuki Nakagawa, Kazue Tsugawa, Atsuko Kitahara, Rie Moriya, Kazuto Takahashi, Yoshikazu Sumitani, Toshio Hosaka, Hayato Kawakami, Seiichi Oyadomari, Hitoshi Ishida

The authors would like to correct Fig 6, as errors were introduced in the preparation of this figure for publication. In panels E, F, and G of Fig 6, the western blots for SCD-1 were improperly enlarged longitudinally. The authors have provided a corrected version of Fig 6 here that also includes the addition of vertical black lines were non-adjacent bands have been spliced together. The authors confirm that these changes do not alter their findings and have provided the underlying blots as Supporting Information.

**Fig 6:**
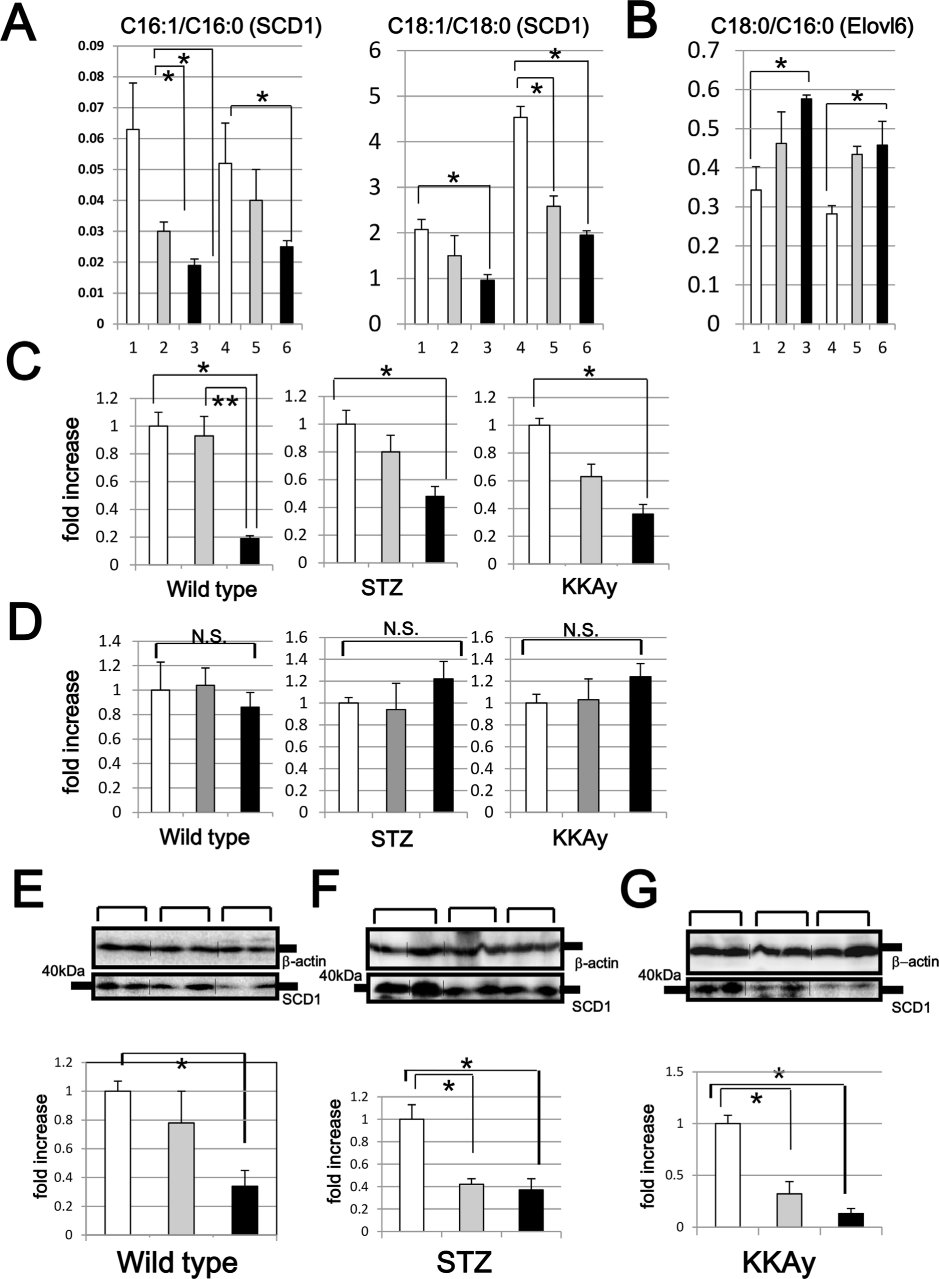
Hepatic SCD1 mRNA and protein levels of wild type, STZ and KKAy mice. The C16:1/C16:0, C18:1/C18:0 (A) and C18:0/C16:0 (B) ratios were calculated, based on data obtained from the serum fatty acid analysis (line 1:WSC, line 2:WLC, line 3:WSR, line 4:KSC, line 5:KLC, line 6:KSR). SCD1 (C) and Elovl6 (D) mRNA levels of all murine models were analyzed by quantitative real time PCR (white bar: mice fed the SC diet, gray bar: mice fed the LC diet, black bar: mice fed the SR diet). Hepatic SCD1 protein levels of wild type (E), STZ (F) and KKAy (G) mice were analyzed by western blotting. In the middle panels, representative data (two samples for each group) are presented. In the lower panels, each column shows the mean ± S.E. obtained from 6 mice (white bar: mice fed the SC diet, gray bar: mice fed the LC diet, black bar: mice fed the SR diet). Upper panels show the internal control using anti-β actin antibody. *p<0.05 (vs corresponding mice fed the SC diet). **p<0.05 (vs corresponding mice fed the SR diet).

## Supporting Information

S1 File Raw blots used to create Fig 6. (PDF)
