# Supplementary figures and images for: Correction: Long-Term Low Carbohydrate Diet Leads to Deleterious Metabolic Manifestations in Diabetic Mice
Source: PLoS One. 2016 May 16;11(5):e0155751. doi: 10.1371/journal.pone.0155751 (PMC4868301; doi:10.1371/journal.pone.0155751)

**Wild type-1(each n=3)**

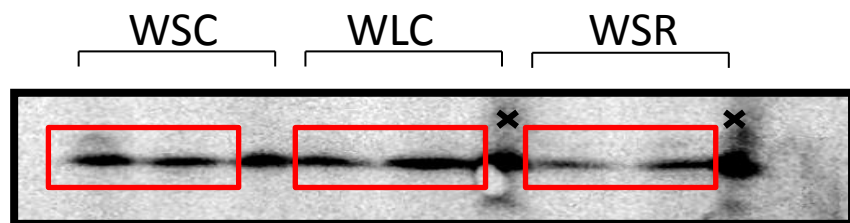

**Wild type-2(each n=3)**

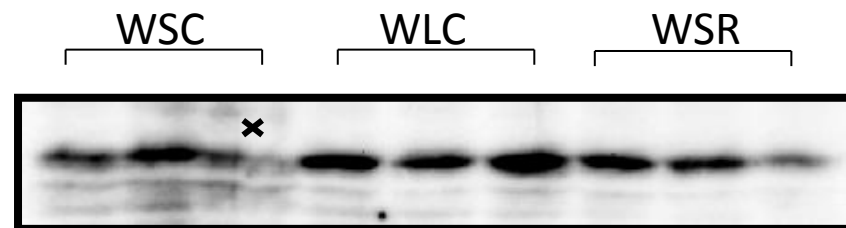

**STZ-1(each n=3)**

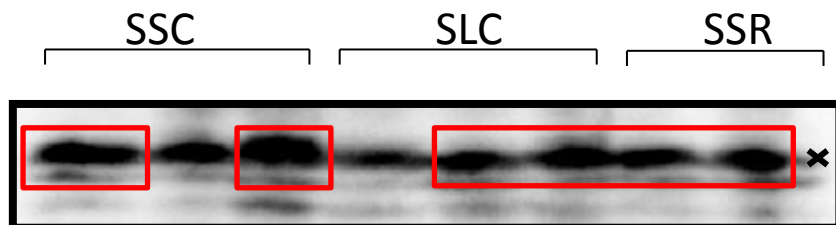

**STZ-2(each n=3)**

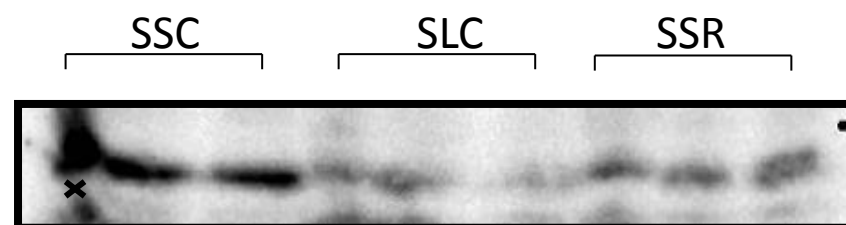

**KKAy-1(each n=3)**

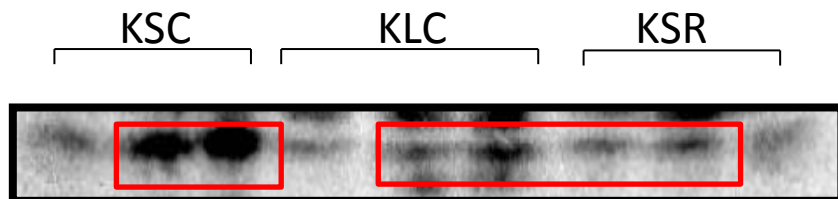

**KKAy-2(each n=3)**

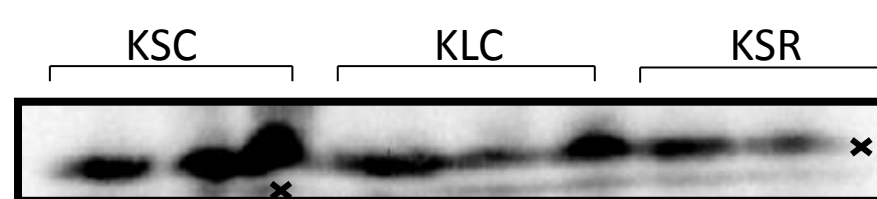

Supplement: S1 File — (PDF) [file pone.0155751.s001.pdf]
